# Biocurators: Contributors to the World of Science

**DOI:** 10.1371/journal.pcbi.0020142

**Published:** 2006-10-27

**Authors:** Philip E Bourne, Johanna McEntyre

Computational biology is a discipline built upon data (mostly free access), found in biological databases, and knowledge (mostly not free access), found in the literature. So important are these online sources of data that the discipline, and indeed this Journal, simply would not exist without them. Whether we are using the data in “browse mode”—doing a PubMed search, looking up a reaction in an enzymatic pathway, or in “compute mode”—analysis of a large dataset, we usually visit Web sites and download information without a second thought. Since our discipline is so dependent on the availability, extent, and quality of biological data, it is worth taking some time to think about the processes of data accessibility, annotation, and validation. These processes depend very much on biocurators—trained staff who ensure the information you are receiving is as complete and accurate as possible.

Biocurators can be considered the museum catalogers of the Internet age: they turn inert and unidentifiable objects (now virtual) into a powerful exhibit from which we can all marvel and learn. That would be a decent enough contribution to the world of science, but the task of the biocurator is even more extensive. Computational biologists do not expect to merely walk through the door, cast a casual eye over the exhibit, and exit wiser (although we frequently do); we also want to add our own data to the exhibit, plus pick and choose pieces of it to take home and create new exhibits of our own. Oh, and we would like to do all these things with minimal effort, please. We can be a pretty exacting bunch of customers, and it takes skills over and above a knowledge of biology to juggle the different needs of data submitters, information seekers, and power players.“We pay homage to these special individuals who are dedicated to making our research endeavors a success.”

In this October issue, we pay homage to these special individuals who are dedicated to making our research endeavors a success. We do so through two Perspectives written by biocurators working with different types of biological data. The first is by biocurators from the Research Collaboratory for Structural Bioinformatics Protein Data Bank (PDB), a well-established biological resource of macromolecular structure data used by more than 10,000 individual scientists per day, and the second by biocurators of the Immune Epitope Database and Analysis Resource (IEDB), a new resource detailing known epitopes and their immunological outcomes. The PDB validates the quality and consistency of primary data submitted by structural biologists as a prerequisite to publication. The IEDB curates the published literature, extracting relevant facts about the epitopes discussed therein. As you read these two Perspectives, similarities and differences concerning the approaches will emerge. But more than anything, we hope you are struck by the level of professionalism and dedication that goes into helping to make the quality research articles that you read in this Journal and elsewhere.

These two articles are told from the perspective of the biocurators themselves. It is only two perspectives; we certainly encourage you to send eLetters with your own perspective on biocuration, either as a curator of a different type of information, or as a person whose information has been curated, or as a consumer of information that has been curated. If you are not moved to comment, at least give a thought to the person upon whose efforts your research may well depend.

## Abbreviations

IEDB: Immune Epitope Database and Analysis Resource
PDB: Research Collaboratory for Structural Bioinformatics Protein Data Bank

